# Younger but sicker? Cohort trends in disease accumulation among middle-aged and older adults in Scotland using health-linked data from the Scottish Longitudinal Study

**DOI:** 10.1093/eurpub/ckae062

**Published:** 2024-04-11

**Authors:** Eloi Ribe, Genevieve Isabelle Cezard, Alan Marshall, Katherine Keenan

**Affiliations:** School of Economic, Social and Political Sciences, University of Southampton, Southampton, UK; British Heart Foundation Cardiovascular Epidemiology Unit, Department of Public Health and Primary Care, University of Cambridge, Cambridge, UK; Victor Phillip Dahdaleh Heart and Lung Research Institute, University of Cambridge, Cambridge, UK; School of Social and Political Science, University of Edinburgh, Edinburgh, UK; School of Geography and Sustainable Development, University of St Andrews, St Andrews, UK

## Abstract

**Background:**

In the United Kingdom, rising prevalence of multimorbidity—the co-occurrence of two or more chronic conditions- is coinciding with stagnation in life expectancy. We investigate patterns of disease accumulation and how they vary by birth cohort, social and environmental inequalities in Scotland, a country which has long suffered from excess mortality and poorer health outcomes relative to its neighbours.

**Methods:**

Using a dataset which links census data from 1991, 2001 and 2011 to disease registers and hospitalization data, we follow cohorts of adults aged 30–69 years for 18 years. We model physical and mental disease accumulation using linear mixed-effects models.

**Results:**

Recent cohorts experience higher levels of chronic disease accumulation compared to their predecessors at the same ages. Moreover, in more recently born cohorts we observe socioeconomic status disparities emerging earlier in the life course, which widen over time and with every successive cohort. Patterns of chronic conditions are also changing, and the most common diseases suffered by later born cohorts are cancer, hypertension, asthma, drug and alcohol problems and depression.

**Conclusion:**

We recommend policies which target prevention of chronic disease in working age adults, considering how and why certain conditions are becoming more prevalent across time and space.

## Introduction

More people suffer from multiple simultaneous chronic diseases, or multimorbidity, during their lifespan than previously,^1^ partly as a result of population ageing. In England, the prevalence of individuals having two or more chronic diseases increased between 2004 and 2019 from 31% to 53%.^2^ Multimorbidity is associated with poorer quality of life and higher incidence of mortality,^3^^,^^4^ and causes profound challenges for health systems, including increased costs and greater levels of unpaid informal care to meet growing gaps in state-funded social care.^1^^,^^5^ Research from several high-income settings suggests an earlier onset and higher prevalence of multimorbidity among younger born cohorts compared to older cohorts at corresponding ages.^6–9^ This study investigates the issue using administrative data from Scotland, where there is high prevalence of multimorbidity, whether this is measured using data from primary care, secondary care or social care receipt.^10–12^ We focus on longitudinal accumulation of multimorbidity, rather than prevalence, to better understand life course trajectories.^13^

Scotland has long been the subject of epidemiological attention due to its comparatively high mortality rates relative to the rest of the United Kingdom and other Western European nations.^14^ In common with other high-income countries,^15^ there has been a downturn in Scottish population health, most evident in stalled life expectancy.^16^ Like the United States,^17^ Scotland has witnessed an increase in cardiovascular mortality and ‘deaths of despair’, which include drug-related deaths.^18^ However, in England and Wales, where stalls in mortality are particularly observable at older ages,^19^ in Scotland, mortality among middle-aged men and those over 65 years contribute most to excess mortality.^18^ Several upstream drivers have been suggested, including austerity measures,^15^^,^^16^ which led to cuts in social welfare and reduced the value of unemployment and disability benefits, and reductions in the provision of social care. Austerity policies are likely to have implications for the onset and accumulation of chronic disease, as well as mortality.

It has been noted before using self-reported cohort data, that younger Scottish cohorts suffer higher multimorbidity than their predecessors,^20^ but the sample size and detail of administrative data linked to census data are needed for a comprehensive picture. Explanations for this pattern are currently unclear, with some suggesting increasing obesity rates as a key contributor.^9^^,^^16^ Early onset cancer rates are also increasing,^21^ which would contribute to disease accumulation in younger cohorts. In one study, adjustment for health behaviours did not explain multimorbidity differentials between cohorts.^20^ It is also unclear whether socioeconomic inequalities, which are stark and apparently widening in Scotland,^22^ affect cohort trends in multimorbidity. Multimorbidity shows a consistent socioeconomic gradient, whether measured cross-sectionally or longitudinally.^2^^,^^23^ Reviews suggest a number of potential behavioural, material and life course mechanisms, which may vary by cohort.^23^^,^^24^ Socioeconomic status operates at multiple scales including local area deprivation, housing and environment and individual human capital (e.g. education), and the effect of these may vary by other factors like gender.

To address this gap, this study aimed to investigate 18-year multimorbidity trajectories of middle-aged and older adults aged 30–69 years using linked census-administrative data in Scotland. We address the following research questions:

- How do disease accumulation trajectories vary according to age, gender and birth cohort?
- Do socio-demographic and socio-economic inequalities differ between birth cohorts?

## Method

### Data and sample

We used data from the Scottish Longitudinal Study (SLS) which links three Scottish censuses (1991, 2001 and 2011) to a range of administrative data sources for a 5.3% sample of the Scottish population.^25^ The SLS was linked to the national diabetes register (covering 1 January 1997 to 31 August 2019) the cancer registry (covering 1 January 1980–31 August 2019), and in-and outpatient hospitalization (SMR01, SMR04 covering 1 January 1997–31 August 2019). We selected a cohort of 124 612 SLS participants who were aged 30–69 years on 1 April 2001. We observe participants from 1 April 2001 (when the census was conducted), until 31 July 2019, or until they die, or exit from the study due to emigration.

### Chronic disease identification

Multimorbidity is widely defined as the co-occurrence of two or more chronic conditions, but there is great variability in how multimorbidity is measured in longitudinal studies,^13^ and incidence is sensitive to the number of conditions.^26^ Using a recently developed consensus list of conditions^27^ we extracted disease presence, order and month of first being recorded for 44 conditions (see list, relevant ICD10 codes and prevalence in Supplementary table S1). Diagnoses from the diabetes register (Types 1 and 2) were also included. Where the same disease was recorded more than once across datasets, we chose the earliest date. The derived outcome is an additive index (total count) of these 44 conditions measured annually at 1 January. For some descriptive analyses, we created a binary version of the index classified into 2+ conditions vs. less. As a robustness check, we used an alternative disease score with fewer conditions (the Charlson index^28^) in sensitivity analyses. In addition, we validated our results by looking at trajectories of multimorbidity for individuals aged 40–59 in 2011, for which we had a longer record of the 44 conditions in the index before follow-up.

### Covariates

Age was measured in years as the difference between birth year of respondent and their age in 2001. Birth cohort was classified into (approximate) 5-year groups: 1931–35, 1936–40, 1941–45, 1946–50, 1951–55, 1956–1960, 1961–65 and 1966–1971. Sex was classified into male/female as recorded in the census 2001. We included a range of socioeconomic indicators at different scales. Individual educational attainment was measured in 2001, classified into no qualifications, low (secondary school level), medium (further education e.g. advanced higher), high (any higher education). Household tenure was measured in 2001 (classified into private renting, social renting and owning property). Finally, we measure area-based social deprivation using the Scottish Index for Multiple Deprivation (SIMD) in 2004,^29^ which measures deprivation in 6976 small areas or data zones and based on seven broad domains: income, employment, education, health, access to services, crime and housing, into five distinctive quintiles.

### Statistical analysis

We transformed the data to a person-year file where each cohort member could contribute a maximum of 19 annual observations. We use an accelerated longitudinal design^30^ and fit a series of linear mixed age-cohort models that predict the score of chronic conditions in 2001 and the subsequent change in chronic conditions between 2001 and 2019 (full model specifications and development are available in the supplementary material). Where the age/period/cohort estimates overlap occur around middle age (50–70 years) we compare birth cohorts at the same ages. The linear mixed effects models account for repeated observations over time for the same individuals, and the final models included age from the beginning of the follow-up period and cohort as fixed effects. We tested different age and cohort specifications (linear, quadratic and cubic), assessing their fit using AIC/BIC values, and present those including age and cohort linear and quadratic polynomial forms.

In nested models, we tested interactions between age, birth cohort and gender. To assess variations in socioeconomic inequalities between cohorts, we also tested interactions between age, birth cohort and the three socioeconomic variables (SIMD, educational level and housing tenure). Final models presented in the results only included statistically significant interaction terms (*P* < 0.05). Where there were significant differences between multimorbidity trajectories by cohort and age, we calculated predicted values of the disease score to draw curves for birth cohorts across age by SIMD quintiles, educational level and housing tenure. To measure and visualize socioeconomic inequalities by birth cohort and age, we used model predicted values and calculated crude and relative differences in these comparing, for instance, the most and least socioeconomically deprived categories. So, for example, we compared the model predicted scores for the most- and least-deprived SIMD quintile at age 40, in the birth cohort born in 1970–74.

### Ethics, data access and disclosure

Ethical approval was obtained from the University Teaching and Research Ethics Committee at the University of St Andrews (reference GG14300). This study was also approved by the SLS Research Board (SLS project number 2018_012) and by the Public Benefit and Privacy Panel for Health and Social Care of NHS Scotland (reference 1819–0093). All analyses were performed in accordance with the relevant SLS guidelines and regulations. Data analysis was conducted in a secure environment, the SLS safe haven, at National Records of Scotland, by named researchers (ER, KK) with appropriate training and clearance. Analyses followed SLS guidelines to ensure the confidentiality of the data. In addition, results were prepared following the SLS statistical disclosure control protocol. Numerators and denominators are presented rounded to the nearest 10 and percentage estimated from rounded numbers.

## Results

### Sample description

Analyses included 122 147 individuals with full records on SIMD, educational qualification and household tenure. At the start of the observation period in 2001, 52% of the sample was female (table 1). Most of the sample had no qualifications or low education (60%), and over 20% had higher education. The majority of the sample (75%) owned their house, and nearly 20% lived in social housing. At baseline, the proportion of the sample with 2 or more diseases increased substantially with age and was higher among older people (55 years and over compared to those <55 years). Men were more likely to have multimorbidity than women. The proportion with 2 or more diseases increases with higher deprivation and was more than twice as high in the most versus least deprived SIMD quintile (5 vs. 2%). Likewise, there was an educational gradient, with those with no education having higher multimorbidity. Those who were living in social rented housing or not paying rent had higher multimorbidity than private renters or house owners.

**Table 1:** Sample description at baseline (2001); individuals aged 30–69

Measured at baseline		*N*	Percent	Disease score Mean	2+ diseases (%)
Sex	Males	58 420	47.8	0.18	3.8
	Females	63 727	52.2	0.14	2.7
Age group	30–35	14 709	12.0	0.05	ND
	35–39	16 730	13.7	0.06	ND
	40–44	18 778	15.4	0.09	1.4
	45–49	16 934	13.9	0.11	2.0
	50–54	17 261	14.1	0.16	3.1
	55–59	14 307	11.7	0.22	4.8
	60–64	12 562	10.3	0.31	6.8
	65–69	10 866	8.9	0.41	9.3
SIMD	(1) Most deprived	21 207	17.4	0.24	5.2
	(2)	23 159	19.0	0.19	4.1
	(3)	24 509	20.1	0.15	3.0
	(4)	25 817	21.1	0.12	2.3
	(5) Least deprived	27 455	22.5	0.11	2.0
Education	No qualifications	45 710	37.4	0.25	5.4
	Low	27 621	22.6	0.12	2.2
	Medium	22 701	18.6	0.10	1.8
	High	26 115	21.4	0.10	1.7
HH Tenure	Owned	91 189	74.7	0.51	2.5
	Private rented	4328	3.5	0.51	2.7
	Social rented	24 322	19.9	0.82	5.9
	No rent	2308	1.9	0.76	5.5
Total		122 147	100.0	0.16	3.2

*Source*: Scottish Longitudinal Study.

*Note:* household tenure missing cases 2465 (1.98%). ND, Not disclosed due to cell size count.

Supplementary table S2 shows the 10 most common diseases to be diagnosed over the observation period 2001–2019, from the 44 conditions in our index, stratified by age group of the individual at baseline. Cancer was the most common disease in every age group. After cancer, hypertension was most common in almost all age groups. Alcohol/drug misuse as a more frequently occurring condition in younger adults (ie those aged <55 years at baseline) than among older adults. Depression and asthma were more commonly diagnosed in younger vs older age groups. On the other hand, among adults aged 50 and over, hypertension, osteoarthritis, coronary heart disease (CHD), diabetes and arrhythmia were among the top 5 diseases to be newly recorded.

### Chronic disease accumulation patterns by age and cohort

We show nested multilevel models in Supplementary table S3 which model the outcome of chronic disease score. Before running these more complex models, we tested the fit of a simple model containing age, gender and cohort, and compared this with one containing an age*cohort interaction. This suggested that a model with age*cohort interactions was a better fit.

**Figure 1:**
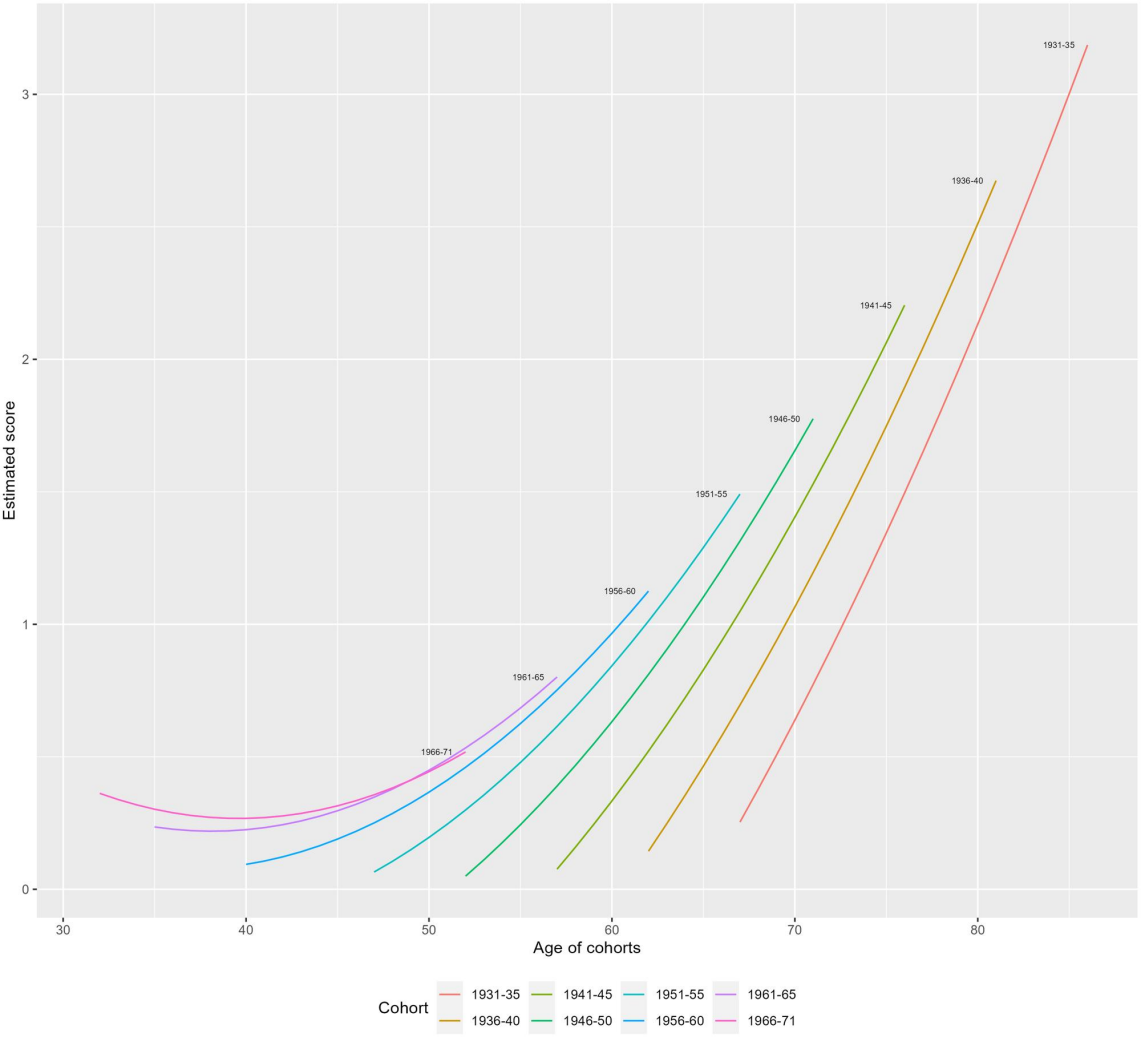
Predicted multimorbidity scores by cohort and age (based on model 1 estimates in Supplementary table S3). Source: Scottish Longitudinal Study.

Model 1 included main effects for cohort, age and gender, and interactions between cohort and gender, and cohort and age. Disease scores increased with time and birth cohort. We used predicted scores derived from model 1 to visualize average cohort trajectories (figure 1). Each curve represents a disease accumulation trajectory for a given cohort, starting in 2001 and tracking the average disease scores over the next 18 years. This demonstrates rapid increases with age, and a somewhat curvilinear pattern at younger ages. Later born birth cohorts also have higher average multimorbidity scores, suggesting that at similar ages, more recently born cohorts suffer higher levels of multimorbidity. For example, at age 50, those born between 1956 and 60 have higher disease scores than the 1951–55 or 1946–50 cohorts at the same age. There is also a statistically significant age-cohort interaction, suggesting steeper age gradients in earlier born cohorts (and at older ages).

## Disparities by socioeconomic factors

### Area-level deprivation

**Figure 2:**
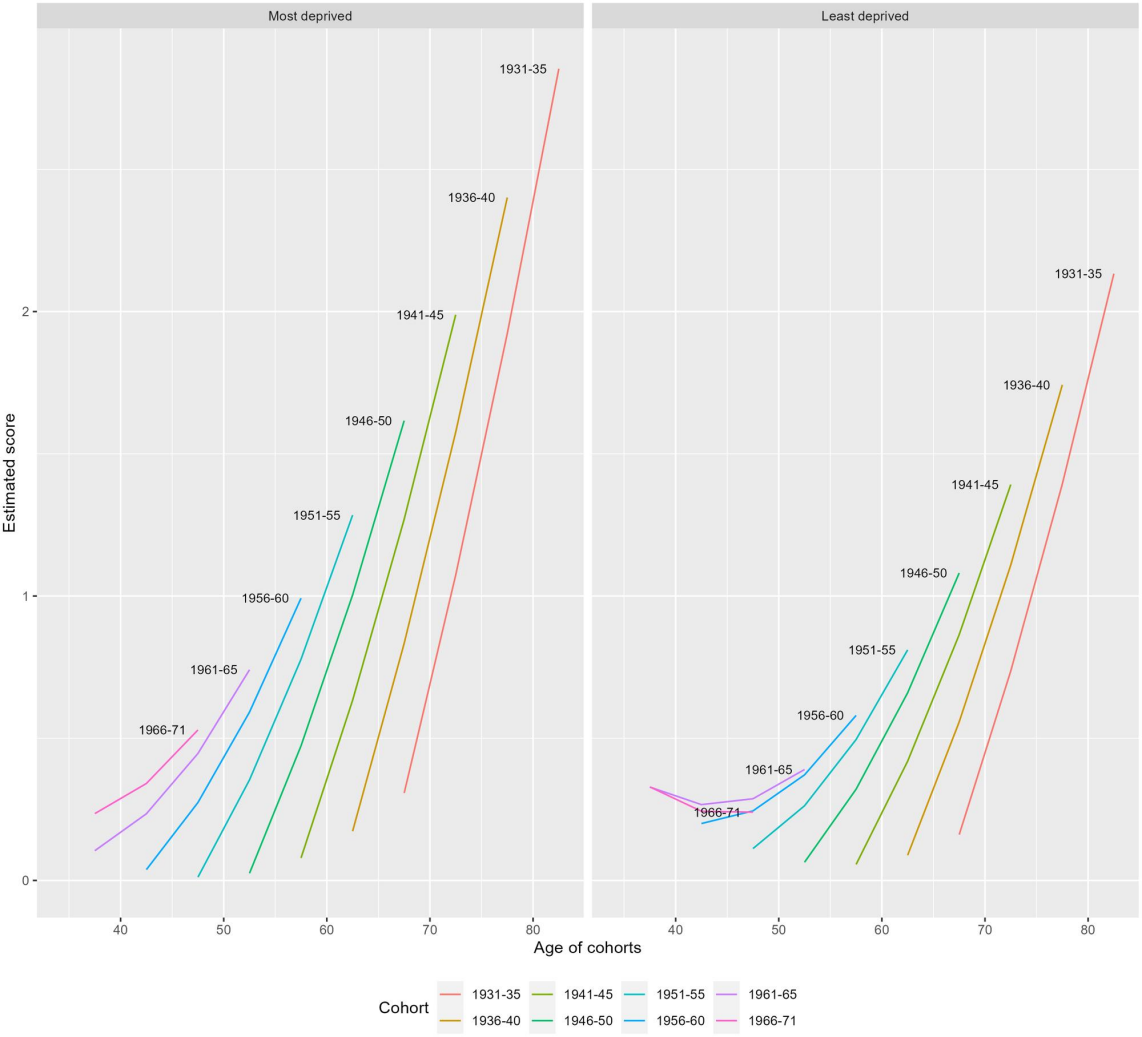
Predicted multimorbidity scores by age, cohort and SIMD quintile. Source: Scottish Longitudinal Study.

Models 2 and 3 in Supplementary table S3 included SIMD quintiles, education and interactions between these variables, age and cohort. Living in more deprived SIMD quintiles is associated with higher average disease scores (model 2). Age significantly interacts with SIMD quintiles, suggesting that those living in more deprived areas have faster disease accumulation. We show predicted disease scores over age and cohort for the most- and least-deprived quintiles in figure 2. The most-deprived group (left) is marked by higher average scores at every age and cohort, and steeper age trajectories within each birth cohort. The average disease trajectory of an individual in the most-deprived SIMD quintile is comparable with that for an individual 5 years older in the least-deprived SIMD quintile, which persists after adjustment for individual education (model 3). At age 40, those in the most-deprived quintile are already starting to see sharp accumulation of conditions with age, whereas 40 year-olds in the least-deprived quintile have flat trajectories.

**Figure 3:**
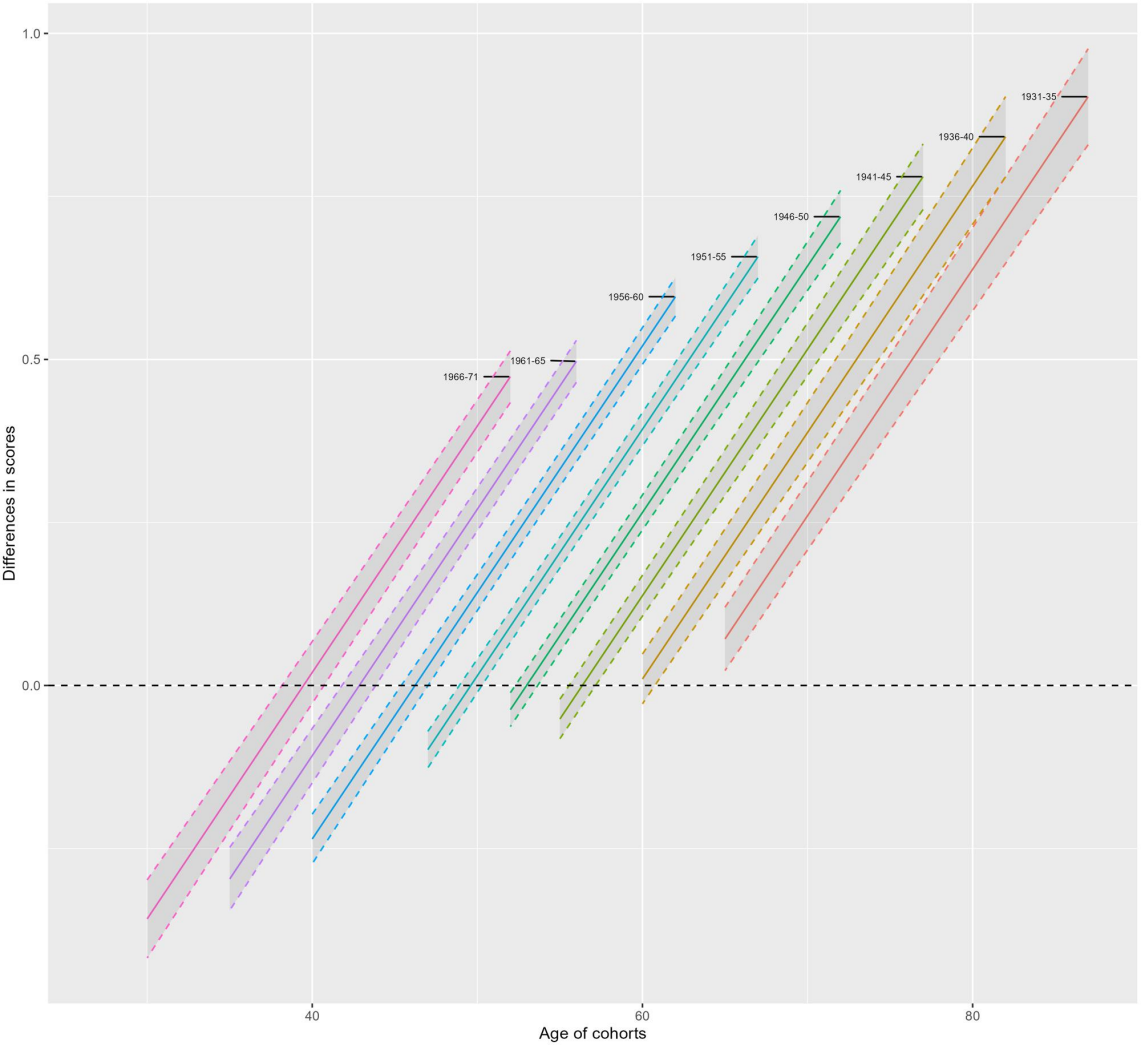
Differences in estimated multimorbidity scores of multimorbidity (A) Least- vs. Most-deprived SIMD quintiles; (B) Social vs. Owned housing tenure; (C) No education vs High) by cohort and age. Source: Scottish Longitudinal Study.

The SIMD by cohort interaction term in models 2 and 3 suggests that SIMD inequalities differ by cohort. To visualize these disparities, we calculated absolute differences in predicted scores between the least-deprived quintile by scores from the most deprived, and plotted these by age and birth cohort (figure 3A). Scores below zero indicate that individuals in the least deprived cohort have higher disease scores in that cohort for a given age; above zero indicates the most-deprived quintile have higher scores. Higher values on the y axis indicate larger absolute disparities in scores between least- and most-deprived quintiles.

At most ages, those living in the most-deprived quintile had higher disease scores. In every cohort, as age increases, so do the score differentials between the least- and most-deprived quintiles, suggesting SIMD inequalities widen with age. Finally, considering overlapping ages for different birth cohorts, the average differences between least- and most-deprived quintile are progressively larger for every cohort born. For example, at age 50, SIMD differentials are higher in the birth cohorts 56–60, and 61–65, than in the 51–55 cohort.

### Disparities by education and household tenure

We observed similar patterns of inequalities in disease scores by education, when adjusting for area-level deprivation (see Supplementary table S3 (Model 3) and Supplementary table S4). The main effect of education in model 3 in Supplementary table S3 shows an inverse relationship between education levels and disease scores, ie people with higher levels of education had lower levels of chronic disease. Supplementary table S4 additionally suggests a statistically significant age and education interaction, meaning that those with high education have slower disease accumulation. The education by cohort interaction term in model 2 suggests widening educational differentials with every subsequent cohort (visualized in figure 3C). Finally, we investigated disparities by household tenure (Supplementary table S5) by running similar models including household tenure, SIMD quintiles, education and interactions between age and cohort. Living in a housing tenure other than privately owned is associated with higher average disease scores, and faster accumulation by age (Supplementary table S5 and Supplementary figure S2). The inequalities are also progressively larger for younger born cohorts.

### Robustness checks

We repeat the modelling with an alternative index of multimorbidity, the Charlson index (Supplementary tables S6–S8), which shows approximately the same pattern of associations but with lower score estimates. We also fitted the same models, restricting to observation period 2011-2019, to allow for more complete historical disease coverage. Supplementary figures S3 and S4 show predicted scores for cohorts over age, and then comparing these by the highest and lowest SIMD quintile. The patterns hold in this restricted sample, suggesting that reduced disease coverage at baseline does not substantially bias our main results.

## Discussion

Our results show that there are persistent social inequalities in the accumulation of chronic disease among working-age adults in Scotland, which widen with age and with every subsequent birth cohort. We also show that social disparities persist across many dimensions and scales, area-level deprivation (SIMD), housing tenure and individual-level human capital (education). Our evidence is based on census-linked administrative data, providing a more robust longitudinal picture than cohort data using self-reported measures.^20^ Our findings corroborate cross-sectional studies which suggest faster accumulation with age, gaps by area-level deprivation and gender.^11^ Taken together with other evidence, it shows that in Scotland, the rest of the UK, the United States and Canada, more recently born cohorts are suffering from complex morbidity at an earlier age compared to later born cohorts.^6^^,^^8^^,^^9^

Our results suggest that in Scotland the time spent in good health in the life course is shortening, a trend noted in other high-income countries.^31^ Drivers of this morbidity expansion have been discussed, for example the role of obesity and metabolic risk factors,^9^^,^^20^ and in the UK, austerity and rising socioeconomic inequalities.^15^ A simple description of the most commonly diagnosed diseases by different ages/cohorts suggests Scottish patterns of disease accumulation are impacted by specific conditions. For example, drug and alcohol use was one of the most common conditions to onset at younger ages. This is not surprising given Scotland’s relatively high rates of drug-related deaths.^18^ Cancer and hypertension are leading diseases at all ages, underlining the need to address systematic risk factors for cardiovascular disease and cancer, such as obesity.

On the other hand, the cohort pattern we observe could be explained by improvements in health care seeking, screening and diagnosis, leading to some diseases have better coverage or being diagnosed at an earlier age. For example, over our study period, the introduction of colorectal screening programmes ^32^ may have increased diagnosis rates, but in socially uneven ways. At the same time government-led initiatives such as the Quality and Outcomes Framework, introduced in 2004 and withdrawn in 2016, which financially incentivized primary care referrals may have led to increased diagnosis rates.^33^ It is reassuring, therefore, that our cohort and age-related patterns are similar to those that have applied methods taking account of age-period-cohort relations.^9^ We also did see any sharp time series breaks in the prevalence of chronic conditions, which would be observed if the pattern were entirely attributable to policy changes. Nevertheless, this deserves further investigation to better understand how much of the increase is due to healthcare system factors.

Younger cohorts also experience wider health disparities, which are expected to widen further with age. This study is unable to pinpoint causal mechanisms and maybe subject to residual confounding, but suggests some important social dimensions which deserve further attention. Uniquely, this study shows large inequalities by housing tenure, showing that living in social housing versus owning your own house, is associated with increasingly deleterious health effects. Importantly, this effect persists after adjustment for SIMD and education, indicating that disadvantage is embedded not only in individual and area-level material deprivation, but patterned by household-level environments, which have so far not been extensively studied. Understanding how chronic disease risk is shaped by household and housing environments is key to reducing inequalities, especially given how younger generations are experiencing much lower levels of housing ownership, and will likely experience greater housing precarity in later life than did previous generations.^34^ Further, the results also suggest attention to subgroups that might be experiencing intersectional disadvantage. For example, men tended to have faster accumulation with age, but this disadvantage is amplified for men living in social housing.

The strengths of this study include the longitudinal data linkage between health administrative data and the census, which enable us to study detailed mental and physical trajectories in a large representative sample of the Scottish population, and to investigate social disparities from a number of dimensions. We also employ multilevel growth curve modelling to tease out age-cohort effects. However, we should mention a few limitations. First, we likely underestimate disease prevalence and incidence because we did not have primary care data. However, our study was not designed to comprehensively estimate prevalence/incidence, but to compare cohorts and socioeconomic groups, which are likely to be comparable. Our disease data does not cover the whole life course of our respondents, meaning some degree of under-coverage which disproportionately affects older individuals. It is therefore reassuring that cohort patterns we observe match those from studies using prospective disease data from cohort studies,^8^^,^^20^ and that our results hold when we estimate follow-up from 2011, which allows better coverage of diseases diagnosed prior to baseline.

Multimorbidity is a complex public health threat for which our health and social care systems are insufficiently prepared. This study highlights worsening trends in Scottish population health which will aggravate the situation. Younger generations typically develop disease at earlier ages than earlier cohorts, and health inequalities, which are already pronounced in Scotland, develop earlier in the life course and continue to widen. This underscores the urgent need for public health policies that tackle the root causes of chronic disease present in environments at multiple scales, which develop early in the life course.

## Supplementary Material

ckae062_Supplementary_Data

## Data Availability

The data underlying this article were provided by the Scottish Longitudinal Study (https://sls.lscs.ac.uk) under licence. Restrictions apply and they are not publicly available. Key points This study investigates 18-year chronic disease trajectories using a representative sample of middle-aged and older Scottish adults, aged 30–69 years, using data which links administrative health care data with census records. Recently born cohorts experience higher levels of chronic disease accumulation compared to their predecessors at the same ages. Socioeconomic status disparities by education, deprivation and housing status are emerging earlier in the life course in more recently born cohorts. Working age adults should be a key target for the prevention of onset of multiple chronic diseases.
