# Supplementary material for: Younger but sicker? Cohort trends in disease accumulation among middle-aged and older adults in Scotland using health-linked data from the Scottish Longitudinal Study
Source: Eur J Public Health. 2024 Apr 11;34(4):696–703. doi: 10.1093/eurpub/ckae062 (PMC11293808; doi:10.1093/eurpub/ckae062)
Supplement: ckae062_Supplementary_Data [file ckae062_supplementary_data.docx]

**Supplementary Materials**

1. **Chronic disease measurement: conditions included**

We adopted the list suggested in ^1^, which contains over 40 conditions. The list of conditions, their corresponding ICD 10 codes (or other ways of identifying) and sample prevalence at the start and end of the observation period are shown below. For conditions in common with Charlson and Elixhauser indexes, we combined codes typically used.

**Table S1: Diseases included, their source and prevalence**

|  | **Disease** | **ICD10 Codes** | **Source** | **Prev 2001** | **Count 2001**  **(N=13,503)** | **Prev 2019** | **Count 2019**  **(N=37,003)** |
| --- | --- | --- | --- | --- | --- | --- | --- |
| 1 | Stroke | G646, G464,I60, I61, I63, I65, I66, I646, I672, I679, I691 | SMR01,04 | 0.39 | 482 | 0.99 | 971 |
| 2 | Coronary artery disease | I21, I22, I252, I25, | SMR01,04 | 0.66 | 823 | 2.69 | 2634 |
| 3 | Heart failure | I11, I13, I50, 'I50',  'I110','I130',I131,  'I132','I255','I420', I142  ,'I429','P290, I1260 | SMR01,04 | 0.45 | 558 | 0.70 | 682 |
| 4 | Peripheral artery disease | 'I70','I71, I73'  'I731','I738','I739',  'I771','I790','I792','K551',  'K558','K559','Z958','Z959' | SMR01,04 | 0.32 | 404 | 0.91 | 897 |
| 5 | Heart valve | 'I05','I06','I07','I08',  'I34','I35','I36',  'I37','I38','I39'  'A520','I091','I098','Q230',  'Q231','Q232','Q233',  'Z952','Z953','Z954' | SMR01,04 | 0.18 | 228 | 0.60 | 592 |
| 6 | Arterial fibrillation (arrhythmia) | 'I47','I48','I49'  'I441','I442','I443','I456',  'I459','R000','R001','R008',  'T821','Z450','Z950' | SMR01,04 | 0.65 | 808 | 2.59 | 2540 |
| 7 | Deep vein thrombosis | I80, I803, I804 | SMR01,04 | 0.02 | 21 | 0.08 | 82 |
| 8 | Aneurysm | I71 | SMR01,04 | 0.06 | 70 | 0.16 | 160 |
| 9 | Hypertension | I10, I11','I12','I13','I15 | SMR01,04 | 0.99 | 1233 | 0.58 | 567 |
| 10 | Diabetes types 1 and 2 | E100','E101','E109','E110',  'E111','E119','E120','E121',  'E129','E130','E131','E139',  'E140','E141','E149  E102','E103','E104','E105',  'E106','E107','E108','E112',  'E113','E114','E115','E116',  'E117','E118', 'E122','E123','E124','E125',  'E126','E127','E128','E132',  'E133','E134','E135','E136',  'E137','E138','E142','E143',  'E144','E145','E146','E147','E148 | SMR01,04, SCI-D | 0.57 | 707 | 2.01 | 1974 |
| 11 | Addison's disease | E271 | SMR01,04 | - | - | 0.01 | 14 |
| 12 | Cystic fibrosis /Bronchiectasis | J47, E84, P75 | SMR01,04 | - | - | - | - |
| 13 | Thyroid conditions | E00','E01','E02','E03  E890, E05 | SMR01,04 | 0.18 | 223 | 1.05 | 1031 |
| 14 | COPD | J40','J41','J42','J43',  'J44','J45','J46','J47  J60','J61','J62','J63',  'J64','J65','J66','J67,  I278','I279','J684',  'J701','J703 | SMR01,04 | 0.49 | 606 | 1.44 | 1415 |
| 15 | Asthma | J45 | SMR01,04 | 0.37 | 456 | 1.93 | 1896 |
| 16 | Parkinson’s | G20, F02.3 | SMR01,04 | - | - | - | - |
| 17 | Epilepsy | G40, G41, F803 | SMR01,04 | 0.16 | 201 | 0.41 | 403 |
| 18 | Multiple/ systematic sclerosis | G35, L940, L941, M348,  M349, M350, G35" | SMR01,04 | 0.07 | 90 | 0.20 | 197 |
| 19 | Paralysis | G81','G82,  G041','G114','G801','G802','  G830','G831','G832','  G833','G834','G839 | SMR01,04 | 0.10 | 130 | 0.25 | 242 |
| 20 | Transient ischemic attack | G45 | SMR01,04 | 0.09 | 106 | 0.40 | 393 |
| 21 | Peripheral neuropathy | G900, G60, G61, G62, G63, G64 | SMR01,04 | 0.05 | 65 | 0.15 | 152 |
| 22 | Chronic pain | R521, R522 | SMR01,04 | 0.02 | 29 | 0.09 | 88 |
| 23 | Cancer  combines all elixhauser codes for lymphoma, metastatic cancer ad cancer | C0-C97 | SMR01,04, 06 | 1.70 | 2113 | 7.61 | 7465 |
| 24 | Dementia | A810, F02, F03, F051, G912 | SMR01,04 | 0.04 | 50 | 0.09 | 84 |
| 25 | Schizophrenia | F062, F20 (exc F204), F230, F231, F232 | SMR01,04 | 0.05 | 67 | 0.13 | 130 |
| 26 | Depression | F32, F33,  F204',F251, | SMR01,04 | 0.27 | 333 | 0.88 | 859 |
| 27 | Anxiety | F064, F40, F41, F606, | SMR01,04 | 0.16 | 198 | 0.67 | 660 |
| 28 | Bipolar disorder | F30, F31 | SMR01,04 | 0.08 | 95 | 0.16 | 153 |
| 30 | Drug or alcohol misuse | E244,F10,  G312,G621,G721,  I426,K292,K700,  K701,K702,K703,  K704,K709,K852,  K860,F102,F112  F122,F132,F142,  F152,F162,F172,  F182,F192" | SMR01,04 | 0.49 | 608 | 1.56 | 1531 |
| 31 | Eating disorder | F50, F983 | SMR01,04 | - | - | - | - |
| 32 | Autism | F84 | SMR01,04 | - | - | 0.01 | 12 |
| 33 | Post-traumatic stress disorder | F431 | SMR01,04 | - | - | 0.01 | 14 |
| 34 | Connective tissue disease | M05','M32','M33','M34','M06, M315','M351','M353','M360 | SMR01,04 | 0.11 | 133 | 0.56 | 550 |
| 35 | Osteoarthritis | M15, M16, M17,  M18, M19, M47 | SMR01,04 | 0.63 | 785 | 4.72 | 4625 |
| 36 | Osteoporosis | M80, M81, M82 | SMR01,04 | 0.03 | 38 | 0.08 | 74 |
| 37 | Gout | M10, M140 | SMR01,04 | 0.07 | 82 | 0.24 | 239 |
| 38 | Liver disease | B18','K73','K74, K700','K701','K702','K703',  'K709','K717','K713','K714'  ,'K715','K760','K762','K763',  'K764','K768','K769','Z944  K70,'I85',K72','K73','K74  I864','I982','K711','K713'  'K715','K717',  'K765','K766','K767' | SMR01,04 | 0.27 | 333 | 0.67 | 654 |
| 39 | Inflammatory bowel disease | K51, M075, M092 | SMR01,04 | 0.08 | 102 | 0.37 | 365 |
| 40 | Chronic pancreatic disease | K860, K861 | SMR01,04 | 0.02 | 30 | 0.07 | 72 |
| 41 | Peptic ulcer | K25','K26','K27','K28 | SMR01,04 | 0.32 | 404 | 0.92 | 902 |
| 42 | Chronic kidney disease  * includes end stage kidney disease | N18','N19, N052','N053' ,  'N054','N055','N056','N057',  'N250','I120','I131','N032'  ,'N033','N034','N035','N036', N183, N184, N185 | SMR01,04 | 0.51 | 641 | 1.02 | 996 |
| 43 | Endometriosis | N80 | SMR01,04 | 0.18 | 226 | 0.64 | 629 |
| 44 | Anaemia | D510 | SMR01,04 | 0.02 | 25 | 0.06 | 59 |

**2.Accelerated longitudinal design – Linear mixed models: technical specification**

The linear mixed models fitted are specified below (equations 1-3) where MM_ij_ indicates the multimorbidity index score of individual i and at time j for i=1…124,612 and j= 1…j_i_; j is the number of repeated occasions for individual i and ranges from 1 to 19 depending on the number of hospitalization instances that an individual has experienced. In the level 1 repeated measures model, each individual’s multimorbidity trajectory is modelled as a function of time (age) only, and directly indicates within individuals’ change trajectories where age_ij_ denotes the time of hospitalization j for individual i and age^2^_ij_ is the quadratic growth rate of the score of multimorbidity. In equation 1, the intercept β_0i_ is composed of two parts: a fixed part gives the mean multimorbidity index score of individual i at age 50 and a random part, U_ij_, the individual deviations from the mean intercept (equation 2). Similarly, the slope (β_1i_) has a fixed part indication the mean slope and random part U_1j_, which represent the individual deviations from the mean slope (equation 3). The time-specific residual term, e_ij_, is assumed to be normally distributed with a mean at zero.${MM}_{ji}= \beta_{0i}+ \beta_{1i}{age}_{j}+ \beta_{2i}{age}_{ji}^{2}+ e_{ij}$ (1)

$\beta_{0i}= \gamma_{00}+ U_{0i}$ (2)

$\beta_{1i}= \gamma_{10}+ U_{1i}$ (3)

Equation 1 has been extended into two other models to include covariates for age, age-squared, cohort and cohort squared, gender, SIMD and education qualification. In short, we allow multimorbidity trajectories for the observed cohorts to vary by gender, SIMD and education in the respective models. Model 2 (equations 4 and 5) and Model 3 (equations 6 and 7).

$\beta_{0i}= \gamma_{00}+ \gamma_{01}{female}_{i}+ \gamma_{02}{cohort}_{i}+ \gamma_{03}{SIMD}_{i}+ \gamma_{04}{female}_{i}{cohort}_{i}+ \gamma_{05}{SIMD}_{i}{cohort}_{i}+ \gamma_{06}{age}_{i}{cohort}_{i}+U_{0i}$ (4)

$\beta_{1i}= \gamma_{10}+ \gamma_{11}{female}_{i}+ \gamma_{12}{cohort}_{i}+ \gamma_{13}{SIMD}_{i}+ \gamma_{14}{female}_{i}{cohort}_{i}+ \gamma_{15}{SIMD}_{i}{cohort}_{i}+ \gamma_{16}{age}_{i}{cohort}_{i}+U_{1i}$ (5)

$\beta_{0i}= \gamma_{00}+ \gamma_{01}{female}_{i}+ \gamma_{02}{cohort}_{i}+ \gamma_{03}{SIMD}_{i} + \gamma_{04}{education}_{i}+ \gamma_{05}{female}_{i}{cohort}_{i}+ \gamma_{06}{SIMD}_{i}{cohort}_{i}+ \gamma_{07}{age}_{i}{cohort}_{i} + \gamma_{08}{SIMD}_{i}{education}_{i}+U_{0i}$ (6)

$\beta_{1i}= \gamma_{10}+ \gamma_{11}{female}_{i}+ \gamma_{12}{cohort}_{i}+ \gamma_{13}{SIMD}_{i}+ \gamma_{14}{education}_{i}+ \gamma_{15}{female}_{i}{cohort}_{i}+ \gamma_{16}{SIMD}_{i}{cohort}_{i}+ \gamma_{17}{age}_{i}{cohort}_{i}+ \gamma_{18}{SIMD}_{i}{education}_{i}+U_{1i}$ (7)

**Table S2: Most commonly occurring diseases newly diagnosed during the 18-year follow-up by age at baseline.**

|  | **Age in years in 2001** | | | | | |
| --- | --- | --- | --- | --- | --- | --- |
| Number of individuals diagnosed, ranked | **30-34** | **35-39** | **40-44** | **45-49** | **50-54** | **55-59** |
|  | (n=13,882) | (n=15, 677) | (n=16,457) | (n=14,439) | (n=13,982) | (n=10,562) |
| 1 | Cancer | Cancer | Cancer | Cancer | Cancer | Cancer |
| 2 | Asthma | Hypertension | Hypertension | Hypertension | Hypertension | Hypertension |
| 3 | Drug/alcohol misuse | Asthma | Osteoarthritis | Osteoarthritis | Osteoarthritis | Osteoarthritis |
| 4 | Hypertension | Osteoarthritis | CHD | CHD | CHD | CHD |
| 5 | Osteoarthritis | Drug/alcohol misuse | Asthma | Diabetes | Arrythmia | Arrythmia |
| 6 | Depression | CHD | Drug/alcohol misuse | Asthma | Diabetes | Diabetes |
| 7 | Endometriosis | Depression | Diabetes | Drug/alcohol misuse | Asthma | COPD |
| 8 | Diabetes | Diabetes | Arrythmia | Arrythmia | COPD | Asthma |
| 9 | Arrythmia | Arrythmia | Depression | Thyroid | Drug/alcohol misuse | Stroke |
| 10 | CHD | Endometriosis | Thyroid | COPD | Thyroid | Drug/alcohol misuse |

*Source: Scottish Longitudinal Study*

Notes: CHD = coronary heart disease COPD = chronic obstructive pulmonary disorder. Red-shaded cells show diseases that are more likely to onset in younger ages/ later born cohorts; blue shaded are more likely to onset at older ages/ in earlier born cohorts; unshaded show no clear age pattern.

**Table S3: Estimated multimorbidity scores from linear multilevel models, adults aged 30-69, Scotland 2001-2019**

| **Disease scores** | **Model 1**  **β (95% CI)** | **Model 2**  **β (95% CI)** | **Model 3**  **β (95% CI)** |
| --- | --- | --- | --- |
| **Age** | 0.069^***^ [0.069,0.070] | 0.091^***^ [0.089,0.092] | 0.091^***^ [0.089,0.092] |
| **Age quadratic** | 0.002^***^ [0.002,0.002] | 0.002^***^ [0.002,0.002] | 0.002^***^ [0.002,0.002] |
| **Birth cohort** | 0.198^***^ [0.194,0.203] | 0.263^***^ [0.255,0.271] | 0.276^***^ [0.267,0.284] |
| **Birth cohort quadratic** | -0.044^***^ [-0.046,-0.043] | -0.045^***^ [-0.046,-0.043] | -0.045^***^ [-0.046,-0.043] |
| **Females (ref. males)** | 0.003 [-0.010,0.016] | -0.061^***^ [-0.091, -0.031] | -0.073^***^ [-0.132,-0.0415] |
| **Females*Cohort** | -0.006 [-0.012,0.000] | -0.008^**^ [-0.014,-0.002] | -0.007^*^ [-0.013,-0.001] |
| **Age*Cohort** | -0.005^***^ [-0.005,-0.004] | -0.005^***^ [-0.005,-0.004] | -0.005^***^ [-0.005,-0.004] |
| **SIMD (most deprived)** |  |  |  |
| *SIMD 2* |  | -0.146^***^[-0.176,-0.115] | -0.148^***^ [-0.186,-0.110] |
| *SIMD 3* |  | -0.214^***^ [-0.244,-0.184] | -0.236^***^ [-0.275,-0.197] |
| *SIMD 4* |  | -0.299^***^ [-0.329,-0.270] | -0.332^***^ [-0.372,-0.291] |
| *SIMD – least deprived* |  | -0.336^***^ [-0.365,-0.306] | -0.376^***^ [-0.420,-0.332] |
| **SIMD (most deprived)*Linear age** |  |  |  |
| *SIMD 2*Linear age* |  | -0.012^***^ [-0.013,-0.010] | -0.012^***^ [-0.013,-0.010] |
| *SIMD 3*Linear age* |  | -0.021^***^ [-0.023,-0.019] | -0.021^***^ [-0.023,-0.019] |
| *SIMD 4*Linear age* |  | -0.030^***^ [-0.032,-0.028] | -0.030^***^ [-0.032,-0.028] |
| *SIMD – least deprived*Linear age* |  | -0.038^***^[-0.040,-0.036] | -0.038^***^ [-0.040,-0.036] |
| **SIMD (most deprived)*Cohort (1931-35)** |  |  |  |
| *SIMD 2*Cohort* |  | -0.036^***^ [-0.046,-0.026] | -0.038^***^ [-0.048,-0.027] |
| *SIMD 3*Cohort* |  | -0.062^***^ [-0.072,-0.052] | -0.068^***^ [-0.078,-0.058] |
| *SIMD 4*Cohort* |  | -0.088^***^ [-0.098,-0.079] | -0.097^***^ [-0.108,-0.087] |
| *SIMD least deprived*Cohort* |  | -0.117^***^ [-0.126,-0.107] | -0.128^***^ [-0.138,-0.118] |
| **SIMD (most deprived)*Male (ref)** |  |  |  |
| *SIMD 2*Females* |  | 0.059^**^ [0.017,0.101] | 0.064^**^ [0.022,0.106] |
| *SIMD 3*Females* |  | 0.054^*^ [0.013,0.096] | 0.063^**^ [0.022,0.104] |
| *SIMD 4*Females* |  | 0.095^***^ [0.055,0.136] | 0.104^***^ [0.063,0.145] |
| *SIMD Least deprived*Females* |  | 0.071^***^ [0.031,0.112] | 0.079^***^ [0.038,0.119] |
| **Education qualification (ref. none)** |  |  |  |
| *Low* |  |  | -0.144^***^ [-0.183,-0.105] |
| *Medium* |  |  | -0.199^***^ [-0.248,-0.149] |
| *High* |  |  | -0.235^***^ [-0.297,-0.173] |
| **SIMD (ref. most deprived)*Education (ref. none)** |  |  |  |
| *SIMD 2*Low* |  |  | 0.037 [-0.017,0.092] |
| *SIMD 2*Medium* |  |  | 0.043 [-0.023,0.109] |
| *SIMD 2*High* |  |  | 0.068 [-0.011,0.146] |
| *SIMD 3*Low* |  |  | 0.078^***^ [0.023,0.133] |
| *SIMD 3*Medium* |  |  | 0.097^**^ [0.033,0.162] |
| *SIMD 3*High* |  |  | 0.137^***^ [0.063,0.212] |
| *SIMD 4*Low* |  |  | 0.118^***^ [0.0617,0.173] |
| *SIMD 4*Medium* |  |  | 0.135^***^ [0.071,0.199] |
| *SIMD 4*High* |  |  | 0.143^***^ [0.070,0.216] |
| *SIMD Least deprived*Low* |  |  | 0.137^***^ [0.085,0.202] |
| *SIMD Least deprived*Medium* |  |  | 0.172^***^ [0.106,0.237] |
| *SIMD Least deprived*High* |  |  | 0.160^***^ [0.087,0.234] |
| Individual | 1.223 [1.213, 1.234] | 1.207 [1.196, 1.217] | 1.203 [1.193, 1.213] |
| Var (age slope) | 0.015 [0.014, 0.016] | 0.014 [0.013, 0.015] | 0.014 [0.013, 0.015] |
| Residual | 0.148 [0.148, 0.148] | 0.148 [0.148, 0.148] | 0.148 [0.148, 0.148] |
| *AIC* | 2982066.8 | 2978892.7 | 2978643.4 |
| *N* | 2,120,400 | 2,120,400 | 2,120,400 |

Source: SLS Longitudinal study

95% confidence intervals in brackets

^*^ *p* < 0.05, ^**^ *p* < 0.01, ^***^ *p* < 0.001

**Table S4. Linear mixed regression models with education as the main socio-economic variable included.**

|  | Model 1 | Model 2 |
| --- | --- | --- |
|  |  |  |
| **Age** | 0.085^***^ [0.084,0.086] | 0.085^***^ [0.084,0.086] |
| **Age quadratic** | 0.002^***^ [0.002,0.002] | 0.002^***^ [0.002,0.002] |
| **Birth cohort** | 0.270^***^ [0.264,0.277] | 0.267^***^ [0.261,0.274] |
| **Birth cohort quadratic** | -0.039^***^ [-0.040,-0.037] | -0.039^***^ [-0.041,-0.038] |
| **Females (ref. males)** | -0.012 [-0.034,0.010] | -0.016 [-0.038,0.005] |
| **Females*Cohort** | -0.008^*^ [-0.014,-0.002] | -0.009^**^ [-0.016,-0.003] |
| **Age*Cohort** | -0.003^***^ [-0.004,-0.003] | -0.003^***^ [-0.004,-0.003] |
| **Education (ref none)** |  |  |
| *Low* | -0.155^***^ [-0.182,-0.129] | -0.099^***^ [-0.142,-0.056] |
| *Medium* | -0.213^***^ [-0.239,-0.187] | -0.137^***^ [-0.188,-0.085] |
| *High* | -0.289^***^ [-0.315,-0.264] | -0.228^***^ [-0.293,-0.163] |
| **Education (none)*Linear age** |  |  |
| Low*Linear age | -0.017^***^ [-0.019,-0.016] | -0.017^***^ [-0.019,-0.016] |
| *Medium*Linear age* | -0.023^***^ [-0.025,-0.021] | -0.023^***^ [-0.025,-0.021] |
| *High*Linear age* | -0.032^***^ [-0.033,-0.030] | -0.032^***^ [-0.033,-0.030] |
| **Education (none)*Cohort (1931-35)** |  |  |
| *Low*Cohort* | -0.063^***^ [-0.071,-0.054] | -0.062^***^ [-0.071,-0.054] |
| *Medium*Cohort* | -0.095^***^ [-0.105,-0.086] | -0.094^***^ [-0.104,-0.085] |
| *High*Cohort* | -0.116^***^ [-0.125,-0.108] | -0.114^***^ [-0.123,-0.106] |
| **Education (none)*Male** |  |  |
| *Low*Females* | -0.014 [-0.049,0.021] | -0.00902 [-0.0440,0.0259] |
| *Medium*Females* | 0.019 [-0.018,0.056] | 0.026 [-0.011,0.064] |
| *High*Females* | 0.047^**^ [0.012,0.082] | 0.049^**^ [0.014,0.084] |
| **SIMD (most deprived)** |  |  |
| *SIMD 2* |  | -0.071^***^ [-0.100,-0.042] |
| *SIMD 3* |  | -0.115^***^ [-0.145,-0.085] |
| *SIMD 4* |  | -0.142^***^ [-0.174,-0.109] |
| *SIMD 5* |  | -0.139^***^ [-0.176,-0.103] |
| **SIMD (most deprived)*Education** |  |  |
| *SIMD 2*Low* |  | -0.022 [-0.074,0.030] |
| *SIMD 2*Medium* |  | -0.026 [-0.089,0.037] |
| *SIMD 2*High* |  | 0.006 [-0.071,0.083] |
| *SIMD 3*Low* |  | -0.034 [-0.087,0.018] |
| *SIMD 3*Medium* |  | -0.034 [-0.096,0.028] |
| *SIMD 3*High* |  | 0.020 [-0.053,0.093] |
| *SIMD 4*Low* |  | -0.043 [-0.096,0.011] |
| *SIMD 4*Medium* |  | -0.054 [-0.115,0.008] |
| *SIMD 4*High* |  | -0.013 [-0.084,0.059] |
| *SIMD 5*Low* |  | -0.072^*^ [-0.129,-0.015] |
| *SIMD 5*Medium* |  | -0.079^*^ [-0.142,-0.016] |
| *SIMD 5*High* |  | -0.048 [-0.121,0.024] |
| Individual | 1.208 [1.197, 1.218] | 1.203 [1.193, 1.213] |
| Var (age slope) | 0.014 [0.014, 0.014] | 0.014 [0.013, 0.014] |
| Residual | 0.148 [0.148, 0.148] | 0.148 [0.148, .0148] |
| *AIC* | 2979272.4 | 2978937.9 |
| *N* | 2,120,400 | 2,120,400 |

Source: SLS longitudinal study

95% confidence intervals in brackets

^*^ *p* < 0.05, ^**^ *p* < 0.01, ^***^ *p* < 0.001

**Figure S1: Differences in** **estimated disease scores, comparing those with no education vs those with higher education, plotted by age and cohort. Based on model 2 in Table S2.**


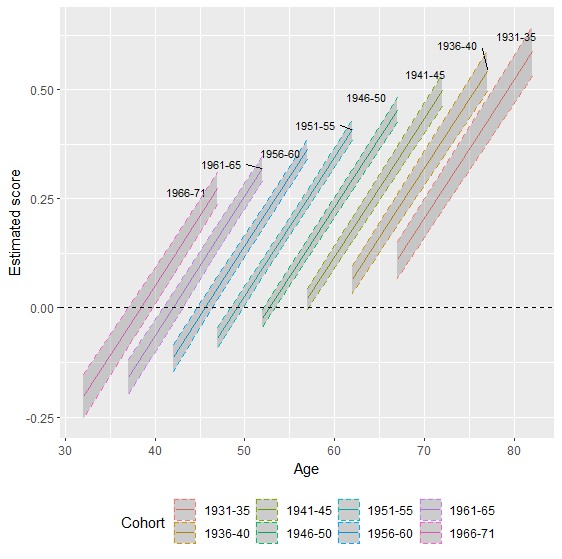


*Source: Scottish Longitudinal Study*

**Table S5. Linear mixed regression models with household tenure as the main socio-economic variable included.**

|  | Model 1 | Model 2 |
| --- | --- | --- |
| **Age** | 0.061^***^ [0.061,0.062] | 0.061^***^ [0.061,0.062] |
| **Age quadratic** | 0.002^***^ [0.002,0.002] | 0.002^***^ [0.002,0.002] |
| **Birth cohort** | 0.175^***^ [0.171,0.180] | 0.177^***^ [0.172,0.182] |
| **Birth cohort quadratic** | -0.044^***^ [-0.046,-0.043] | -0.045^***^ [-0.046,-0.043] |
| **Females (ref. males)** | 0.011 [-0.003,0.026] | 0.009 [-0.005,0.024] |
| **Females*Cohort** | -0.011^***^ [-0.003,-0.026] | -0.011^***^ [-0.017,-0.005] |
| **Age*Cohort** | -0.005^***^ [-0.005,-0.004] | -0.005^***^ [-0.005,-0.004] |
| **Household tenure (ref owned)** |  |  |
| *Private rent* | 0.098^***^ [0.049,0.147] | 0.206^***^ [0.136,0.276] |
| *Social rent* | 0.381^***^ [0.358,0.405] | 0.376^***^ [0.346,0.406] |
| *Rent free* | 0.280^***^ [0.211,0.349] | 0.341^***^[0.255,0.427] |
| **Household tenure (ref owned)*Linear age** |  |  |
| *Private rent*Linear age* | 0.015^***^ [0.012,0.018] | 0.0151^***^ [0.0119,0.0183] |
| *Social rent*Linear age* | 0.035^***^ [0.034,0.036] | 0.0350^***^ [0.0335,0.0364] |
| *Rent free*Linear age* | 0.030^***^ [0.025,0.034] | 0.0297^***^ [0.0253,0.0340] |
| **Household tenure (ref owned)*Cohort** |  |  |
| *Private rent*Cohort* | 0.059^***^ [0.042,0.076] | 0.072^***^ [0.054,0.090] |
| *Social rent*Cohort* | 0.108^***^ [0.101,0.115] | 0.116^***^ [0.108,0.124] |
| *Rent free*Cohort* | 0.125^***^ [0.104,0.146] | 0.136^***^ [0.113,0.158] |
| **Household tenure*Males** |  |  |
| *Private rent*Females* | -0.007 [-0.076,0.062] | -0.013 [-0.082,0.056] |
| *Social rent*Females* | -0.095^***^ [-0.127,-0.064] | -0.099^***^ [-0.131,-0.067] |
| *Rent free*Females* | -0.067 [-0.161,0.026] | -0.091 [-0.185,0.002] |
| **SIMD (most deprived)** |  |  |
| *SIMD 2* |  | -0.036^***^ [-0.057,-0.015] |
| *SIMD 3* |  | -0.0630^***^ [-0.084,-0.041] |
| *SIMD 4* |  | -0.088^***^ [-0.110,-0.066] |
| *SIMD 5* |  | -0.104^***^ [-0.127,-0.082] |
| **Education (ref none)** |  |  |
| *Low* |  | -0.0095 [-0.031,0.012] |
| *Medium* |  | -0.027^*^ [-0.049,-0.005] |
| *High* |  | -0.053^***^ [-0.074,-0.033] |
| **Household tenure (ref owned)*Education** |  |  |
| *Private rent*Low* |  | -0.105^*^ [-0.204,-0.006] |
| *Private rent*Medium* |  | -0.200^***^ [-0.300,-0.099] |
| *Private rent*High* |  | -0.215^***^ [-0.309,-0.120] |
| *Social rent*Low* |  | -0.124^***^ [-0.166,-0.082] |
| *Social rent*Medium* |  | -0.132^***^ [-0.185,-0.078] |
| *Social rent*High* |  | -0.0957^**^ [-0.166,-0.026] |
| *Rent free*Low* |  | -0.141^*^ [-0.270,-0.011] |
| *Rent free*Medium* |  | -0.250^**^ [-0.402,-0.098] |
| *Rent free*High* |  | -0.141^*^ [-0.280,-0.003] |
| Individual | 1.198 [1.188,1.209] | 1.194 [1.184,1.205] |
| Var (age slope) | 0.014 [0.013,0.014] | 0.013 [0.012,0.014] |
| Residual | 0.148 [0.148,0.148] | 0.148 [0.148,0.148] |
| *AIC* | 2977882.8 | 2977596.0 |
| *N* | 2120400 | 2120400 |

Source: SLS longitudinal study

95% confidence intervals in brackets

^*^ *p* < 0.05, ^**^ *p* < 0.01, ^***^ *p* < 0.001

**Figure S2: Predicted disease scores by cohort and household tenure across age in 2001**


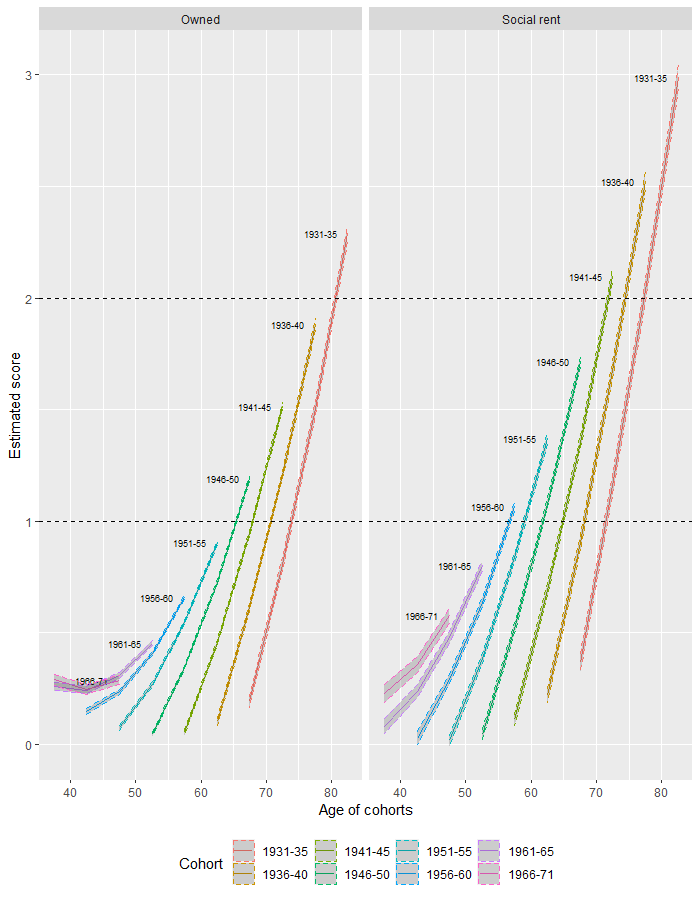


## **Table S6: Estimated Charlson multimorbidity scores from linear multilevel models, adults aged 30-69, Scotland 2001-2019, with a focus on SIMD**

|  | Model 1 | Model 2 | Model 3 |
| --- | --- | --- | --- |
| **Age** | 0.051^***^ [0.050,0.051] | 0.069^***^ [0.067,0.070] | 0.069^***^ [0.067,0.070] |
| **Age quadratic** | 0.002^***^ [0.002,0.002] | 0.002^***^ [0.002,0.002] | 0.002^***^ [0.002,0.002] |
| **Birth cohort** | 0.151^***^ [0.148,0.155] | 0.198^***^ [0.191,0.204] | 0.206^***^ [0.199,0.213] |
| **Birth cohort quadratic** | -0.029^***^ [-0.030,-0.028] | -0.030^***^ [-0.031,-0.028] | -0.029^***^ [-0.031,-0.028] |
| **Females (ref. males)** | 0.010 [-0.001,0.021] | -0.038^**^ [-0.063,-0.012] | -0.045^***^ [-0.070,-0.019] |
| **Females*Cohort** | -0.019^***^ [-0.024,-0.014] | -0.021^***^ [-0.026,-0.016] | -0.020^***^ [-0.025,-0.015] |
| **Age*Cohort** | -0.002^***^ [-0.002,-0.002] | -0.002^***^ [-0.002,-0.002] | -0.002^***^ [-0.002,-0.002] |
| **SIMD (most deprived)** |  |  |  |
| *SIMD 2* |  | -0.096^***^ [-0.122,-0.071] | -0.094^***^ [-0.126,-0.063] |
| *SIMD 3* |  | -0.133^***^ [-0.159,-0.108] | -0.147^***^ [-0.179,-0.114] |
| *SIMD 4* |  | -0.181^***^ [-0.206,-0.157] | -0.198^***^ [-0.231,-0.164] |
| *SIMD – least deprived* |  | -0.191^***^ [-0.216,-0.167] | -0.210^***^ [-0.246,-0.173] |
| **SIMD (most deprived)*Linear age** |  |  |  |
| *SIMD 2*Linear age* |  | -0.010^***^ [-0.011,-0.008] | -0.010^***^ [-0.011,-0.008] |
| *SIMD 3*Linear age* |  | -0.018^***^ [-0.020,-0.016] | -0.018^***^ [-0.020,-0.016] |
| *SIMD 4*Linear age* |  | -0.025^***^ [-0.027,-0.023] | -0.025^***^ [-0.027,-0.023] |
| *SIMD – least deprived*Linear age* |  | -0.031^***^ [-0.032,-0.029] | -0.031^***^ [-0.032,-0.029] |
| **SIMD (most deprived)*Cohort (1931-35)** |  |  |  |
| *SIMD 2*Cohort* |  | -0.024^***^ [-0.032,-0.016] | -0.024^***^ [-0.033,-0.016] |
| *SIMD 3*Cohort* |  | -0.044^***^ [-0.052,-0.036] | -0.048^***^ [-0.057,-0.039] |
| *SIMD 4*Cohort* |  | -0.064^***^ [-0.072,-0.056] | -0.069^***^ [-0.078,-0.061] |
| *SIMD least deprived*Cohort* |  | -0.086^***^ [-0.094,-0.078] | -0.092^***^ [-0.101,-0.084] |
| **SIMD (most deprived)*Male (ref)** |  |  |  |
| *SIMD 2*Females* |  | 0.043^*^ [0.007,0.078] | 0.046^*^ [0.011,0.081] |
| *SIMD 3*Females* |  | 0.044^*^ [0.009,0.078] | 0.051^**^ [0.015,0.085] |
| *SIMD 4*Females* |  | 0.075^***^ [0.040,0.109] | 0.081^***^ [0.047,0.116] |
| *SIMD Least deprived*Females* |  | 0.051^**^ [0.017,0.085] | 0.055^**^ [0.021,0.089] |
| **Education qualification (ref. none)** |  |  |  |
| *Low* |  |  | -0.086^***^ [-0.119,-0.053] |
| *Medium* |  |  | -0.126^***^ [-0.167,-0.085] |
| *High* |  |  | -0.153^***^ [-0.206,-0.101] |
| **SIMD (ref. most deprived)*Education (ref. none)** |  |  |  |
| *SIMD 2*Low* |  |  | 0.008 [-0.037,0.054] |
| *SIMD 2*Medium* |  |  | 0.022 [-0.033,0.077] |
| *SIMD 2*High* |  |  | 0.054 [-0.011,0.120] |
| *SIMD 3*Low* |  |  | 0.033 [-0.013,0.079] |
| *SIMD 3*Medium* |  |  | 0.079^**^ [0.025,0.133] |
| *SIMD 3*High* |  |  | 0.086^**^ [0.024,0.149] |
| *SIMD 4*Low* |  |  | 0.048^*^ [0.001,0.095] |
| *SIMD 4*Medium* |  |  | 0.091^***^ [0.037,0.145] |
| *SIMD 4*High* |  |  | 0.095^**^ [0.033,0.156] |
| *SIMD Least deprived*Low* |  |  | 0.081^**^ [0.031,0.130] |
| *SIMD Least deprived*Medium* |  |  | 0.104^***^ [0.049,0.159] |
| *SIMD Least deprived*High* |  |  | 0.094^**^ [0.033,0.156] |
| Individual | -2.410***[-2.415,-2.406] | -2.417***[-2.422,-2.413] | -2.417***[-2.422,-2.413] |
| Var (age slope) | 0.050***[0.043,0.056] | 0.048***[0.041,0.054] | 0.045***[0.039,0.052] |
| Residual | -1.055***[-1.056,-1.054] | -1.055***[-1.056,-1.054] | -1.055***[-1.056,-1.054] |
| *AIC* | 2533719.6 | 2531279.2 | 2531142.1 |
| *N* | 2120400 | 2120400 | 2120400 |

Source: SLS longitudinal study

95% confidence intervals in brackets

^*^ *p* < 0.05, ^**^ *p* < 0.01, ^***^ *p* < 0.001

## **Table S7: Estimated Charlson multimorbidity scores from linear multilevel models, adults aged 30-69, Scotland 2001-2019, with a focus on education level**

|  | Model 1 | Model 2 |
| --- | --- | --- |
| **Age** | 0.085^***^ [0.084,0.086] | 0.085^***^ [0.084,0.086] |
| **Age quadratic** | 0.002^***^ [0.002,0.002] | 0.002^***^ [0.002,0.002] |
| **Birth cohort** | 0.270^***^ [0.264,0.277] | 0.267^***^ [0.261,0.274] |
| **Birth cohort quadratic** | -0.039^***^ [-0.041,-0.037] | -0.039^***^ [-0.041,-0.038] |
| **Females (ref. males)** | -0.012 [-0.034,0.010] | -0.016 [-0.038,0.005] |
| **Females*Cohort** | -0.008^*^ [-0.014,-0.002] | -0.010^**^ [-0.016,-0.003] |
| **Age*Cohort** | -0.003^***^ [-0.004,-0.003] | -0.003^***^ [-0.004,-0.003] |
| **Education (ref none)** |  |  |
| *Low* | -0.155^***^ [-0.182,-0.129] | -0.0992^***^ [-0.142,-0.056] |
| *Medium* | -0.213^***^ [-0.239,-0.187] | -0.137^***^ [-0.188,-0.085] |
| *High* | -0.289^***^ [-0.315,-0.264] | -0.228^***^ [-0.293,-0.163] |
| **Education (none)*Linear age** |  |  |
| Low*Linear age | -0.017^***^ [-0.019,-0.016] | -0.017^***^ [-0.019,-0.016] |
| *Medium*Linear age* | -0.023^***^ [-0.025,-0.021] | -0.023^***^ [-0.025,-0.021] |
| *High*Linear age* | -0.032^***^ [-0.033,-0.030] | -0.032^***^ [-0.033,-0.030] |
| **Education (none)*Cohort (1931-35)** |  |  |
| *Low*Cohort* | -0.063^***^ [-0.071,-0.054] | -0.062^***^ [-0.071,-0.054] |
| *Medium*Cohort* | -0.095^***^ [-0.105,-0.086] | -0.094^***^ [-0.104,-0.085] |
| *High*Cohort* | -0.116^***^ [-0.125,-0.108] | -0.114^***^ [-0.123,-0.106] |
| **Education (none)*Male** |  |  |
| *Low*Females* | -0.014 [-0.049,0.021] | -0.009 [-0.044,0.026] |
| *Medium*Females* | 0.019 [-0.018,0.056] | 0.026 [-0.010,0.064] |
| *High*Females* | 0.046^**^ [0.012,0.082] | 0.047^**^ [0.014,0.083] |
| **SIMD (most deprived)** |  |  |
| *SIMD 2* |  | -0.071^***^ [-0.100,-0.042] |
| *SIMD 3* |  | -0.115^***^ [-0.145,-0.085] |
| *SIMD 4* |  | -0.142^***^ [-0.174,-0.109] |
| *SIMD 5* |  | -0.139^***^ [-0.176,-0.103] |
| **SIMD (most deprived)** |  |  |
| *SIMD 2*Low* |  | -0.022 [-0.074,0.030] |
| *SIMD 2*Medium* |  | -0.026 [-0.090,0.037] |
| *SIMD 2*High* |  | 0.006 [-0.071,0.083] |
| *SIMD 3*Low* |  | -0.034 [-0.087,0.018] |
| *SIMD 3*Medium* |  | -0.034 [-0.096,0.028] |
| *SIMD 3*High* |  | 0.020 [-0.053,0.094] |
| *SIMD 4*Low* |  | -0.043 [-0.096,0.011] |
| *SIMD 4*Medium* |  | -0.054 [-0.115,0.008] |
| *SIMD 4*High* |  | -0.013 [-0.085,0.060] |
| *SIMD 5*Low* |  | -0.072^*^ [-0.129,-0.015] |
| *SIMD 5*Medium* |  | -0.079^*^ [-0.142,-0.016] |
| *SIMD 5*High* |  | -0.048 [-0.121,0.024] |
| Individual | 0.831 [0.823, 0.840] | 0.829 [0.822, 0.837] |
| Var (age slope) | 0.004 [0.003, 0.004] | 0.004 [0.003, 0.004] |
| Residual | 0.121 [0.120, 0.121] | 0.121 [0.121, 0.121] |
| *AIC* | 2979272.4 | 2978937.9 |
| *N* | 2120400 | 2120400 |

Source: SLS longitudinal study

95% confidence intervals in brackets

^*^ *p* < 0.05, ^**^ *p* < 0.01, ^***^ *p* < 0.001

## **Table S8: Estimated Charlson multimorbidity scores from linear multilevel models, adults aged 30-69, Scotland 2001-2019, with a focus on household tenure**

|  | Model 1 | Model 2 |
| --- | --- | --- |
| **Age** | 0.045^***^ [0.044,0.045] | 0.045^***^ [0.044,0.045] |
| **Age quadratic** | 0.0016^***^ [0.002,0.002] | 0.002^***^ [0.002,0.002] |
| **Birth cohort** | 0.137^***^ [0.133,0.141] | 0.138^***^ [0.134,0.142] |
| **Birth cohort quadratic** | -0.029^***^ [-0.031,-0.028] | -0.030^***^ [-0.031,-0.028] |
| **Females (ref. males)** | 0.016^**^ [0.004,0.028] | 0.015^*^ [0.003,0.027] |
| **Females*Cohort** | -0.022^***^ [-0.027,-0.017] | -0.022^***^ [-0.027,-0.017] |
| **Age*Cohort** | -0.002^***^ [-0.002,-0.001] | -0.002^***^ [-0.002,-0.002] |
| **Household tenure (ref owned)** |  |  |
| *Private rent* | 0.050^*^ [0.009,0.091] | 0.125^***^ [0.066,0.184] |
| *Social rent* | 0.214^***^ [0.195,0.234] | 0.215^***^ [0.190,0.240] |
| *Rent free* | 0.150^***^ [0.092,0.208] | 0.175^***^ [0.103,0.247] |
| **Household tenure (ref owned)*Linear age** |  |  |
| *Private rent*Linear age* | 0.010^***^ [0.007,0.013] | 0.010^***^ [0.007,0.013] |
| *Social rent*Linear age* | 0.028^***^ [0.027,0.029] | 0.028^***^ [0.027,0.029] |
| *Rent free*Linear age* | 0.023^***^ [0.019,0.027] | 0.023^***^ [0.019,0.026] |
| **Household tenure (ref owned)*Cohort** |  |  |
| *Private rent*Cohort* | 0.029^***^ [0.015,0.043] | 0.038^***^ [0.023,0.053] |
| *Social rent*Cohort* | 0.071^***^ [0.065,0.077] | 0.076^***^ [0.070,0.083] |
| *Rent free*Cohort* | 0.079^***^ [0.061,0.097] | 0.084^***^ [0.065,0.103] |
| **Household tenure*Males** |  |  |
| *Private rent*Females* | -0.021 [-0.078,0.037] | -0.025 [-0.083,0.033] |
| *Social rent*Females* | -0.059^***^ [-0.086,-0.033] | -0.062^***^ [-0.089,-0.035] |
| *Rent free*Females* | -0.058 [-0.14,0.020] | -0.072 [-0.150,0.007] |
| **SIMD (most deprived)** |  |  |
| *SIMD 2* |  | -0.031^***^ [-0.049,-0.013] |
| *SIMD 3* |  | -0.044^***^ [-0.062,-0.026] |
| *SIMD 4* |  | -0.057^***^ [-0.075,-0.038] |
| *SIMD 5* |  | -0.062^***^ [-0.081,-0.043] |
| **Education (ref none)** |  |  |
| *Low* |  | -0.011 [-0.029,0.006] |
| *Medium* |  | -0.015 [-0.033,0.003] |
| *High* |  | -0.038^***^ [-0.055,-0.020] |
| **Household tenure (ref owned)*Education** |  |  |
| *Private rent*Low* |  | -0.075 [-0.158,0.008] |
| *Private rent*Medium* |  | -0.152^***^ [-0.237,-0.068] |
| *Private rent*High* |  | -0.133^**^[-0.212,-0.054] |
| *Social rent*Low* |  | -0.094^***^ [-0.130,-0.059] |
| *Social rent*Medium* |  | -0.072^**^ [-0.116,-0.027] |
| *Social rent*High* |  | -0.076^*^ [-0.135,-0.018] |
| *Rent free*Low* |  | -0.057 [-0.165,0.052] |
| *Rent free*Medium* |  | -0.149^*^ [-0.277,-0.021] |
| *Rent free*High* |  | -0.051 [-0.167,0.066] |
| Individual | -2.418*** [-2.422,-2.413] | -2.418*** [-2.422,-2.413] |
| Var (age slope) | 0.045*** [0.038,0.051] | 0.041*** [0.035,0.048] |
| Residual | -1.055*** [-1.056,-1.054] | -1.055*** [-1.056,-1.054] |
| *AIC* | 2530902.3 | 2530748.4 |
| *N* | 2120400 | 2120400 |

Source: SLS longitudinal study

95% confidence intervals in brackets

^*^ *p* < 0.05, ^**^ *p* < 0.01, ^***^ *p* < 0.001

**Figure S3: Predicted disease scores by cohort across time (40+) in 2011**


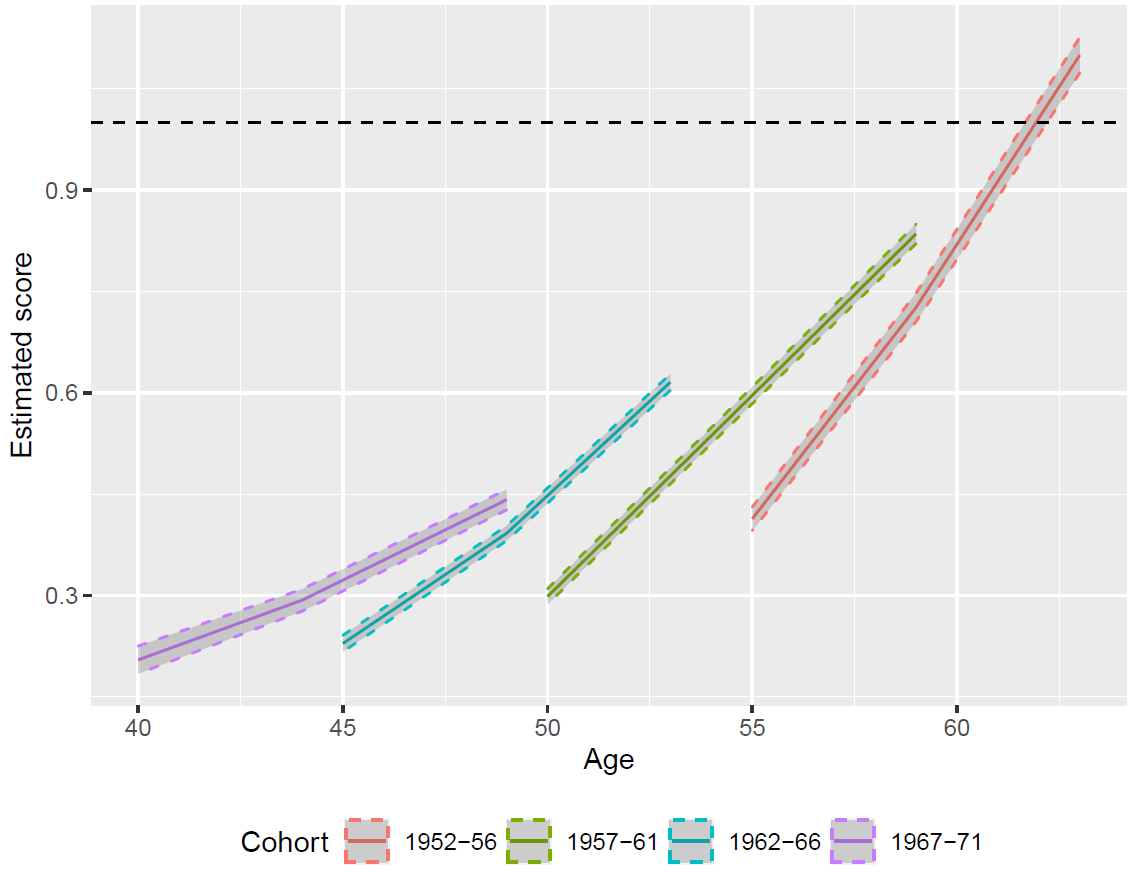


**Fig S4: Predicted disease scores by cohort and SIMD across time (least vs most deprived) (40+) in 2011**


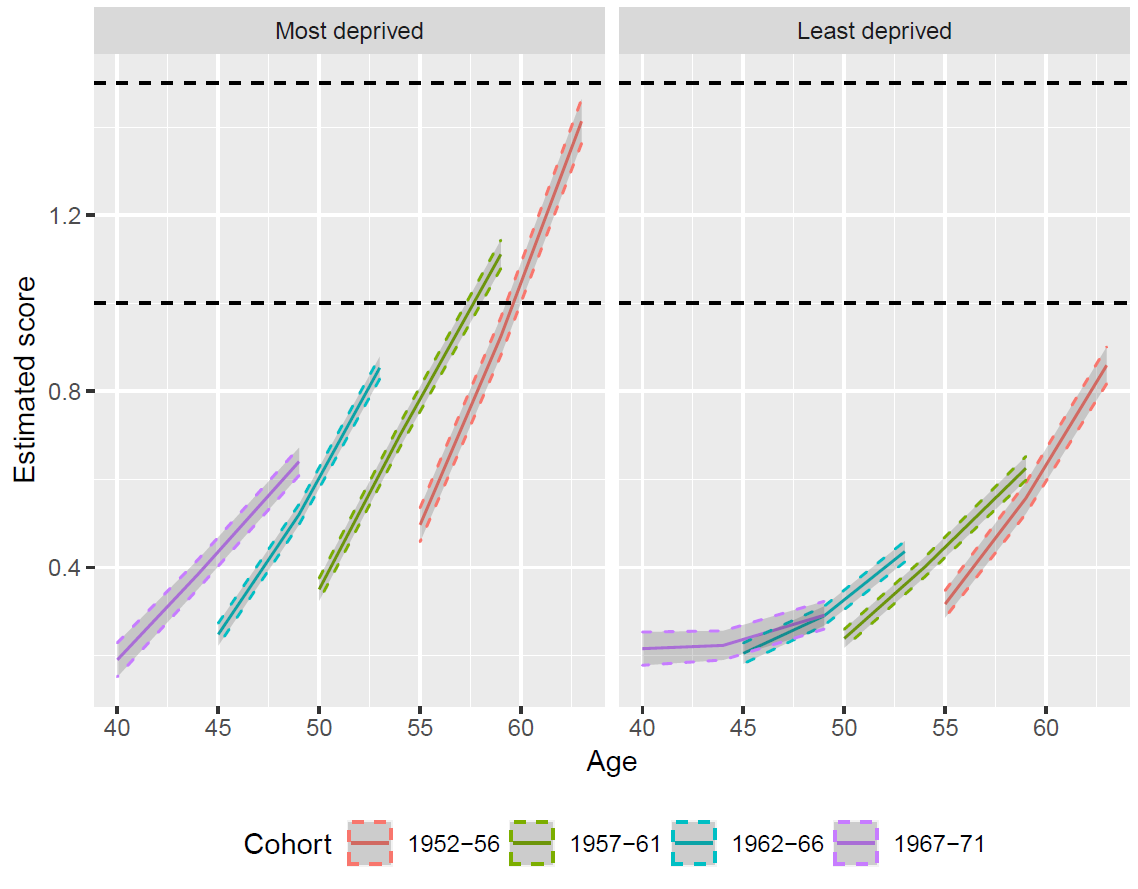


**References**

1 Ho ISS, Azcoaga-Lorenzo A, Akbari A, *et al.* Measuring multimorbidity in research: Delphi consensus study. *BMJ Medicine* 2022; **1**. DOI:10.1136/bmjmed-2022-000247.
